# Coordination Behavior of 3-Ethoxycarbonyltetronic Acid towards Cu(II) and Co(II) Metal Ions

**DOI:** 10.1155/2008/547915

**Published:** 2009-02-03

**Authors:** Giorgos Athanasellis, Georgia Zahariou, Stefanos Kikionis, Olga Igglessi-Markopoulou, John Markopoulos

**Affiliations:** ^1^Laboratory of Organic Chemistry, School of Chemical Engineering, National Technical University of Athens, Zografou Campus, 15773 Athens, Greece; ^2^Institute of Materials Science, NCSR ‘Demokritos’, 15310 Aghia Paraskevi Attikis, Greece; ^3^Laboratory of Inorganic Chemistry, Department of Chemistry, University of Athens, Panepistimiopolis, 15771 Athens, Greece

## Abstract

Tetronic acids, 4-hydroxy-5H-furan-2-ones, constitute a class of heterocyclic compounds with potent biological and pharmacological activity. The *β*, *β*′-tricarbonyl moiety plays an integral role in biological systems and forms a variety of metal complexes. In this report, we present the complexation reactions of 3-ethoxycarbonyl tetronic acids with acetates and chlorides of Cu(II) and Co(II). These complexes have been studied by means of EPR spectroscopy and magnetic susceptibility measurements. From the obtained results, a preliminary complexation mode of the ligand is proposed.

## 1. INTRODUCTION

The chemistry of tetronic acids is a field of continuing interest. The appreciable number of tetronic acids found in nature [1, 2] and their very promising biological activities [3–5] prompted many research groups to attempt new methods for the synthesis of this class of heterocyclic compounds. Tetronic acids and their derivatives are present in a large number of natural products which exhibit a variety of biological and pharmacological properties. This class of heterocyclic compounds includes agglomerins A-D, ylidene tetronic acids [6], and the ATP-ase gastric inhibitors A88696C and A88696F [7], as well as aspertetronins and gregatins isolated from fungi which exhibit antibacterial and antifungal activities [8, 9].

The recent literature gives us a few examples of tetronic acids, both those isolated from nature and those synthesized in the laboratory. Such compounds are the CCK-B receptor antagonist tetronothiodin [10], the marine furanosesterpene natural product (18S)-variabilin [11], and the antibiotic abyssomicin C [12–14].

The coordination chemistry of tetronic acids has been investigated by many research groups in the past. Studies of complexes of oximidobenzotetronic acid complexed with Fe(II), Co(II), Ni(II), Cu(II), Zn(II), Cd(II), and U(VI) by conductometric and by pH-metric titrations revealed that the metals form 1:2 (metal:ligand) complexes with the exception of Fe(II) and Co(II) complexes which form 1:3 ratios [15]. Processes for the synthesis of a Pt(II) complex with a 3-acetyl tetronic acid [16] and Pd(II) complexes involving tetronic acid derivatives [17] have been reported. Furthermore, a series of 3-acyl tetronic acids and their Cu(II) complexes, which possess a tricarbonylmethane structure, were prepared and tested for antimicrobial activity [18]. In addition, 3-(1-iminoalkyl) tetronic acids and their Cu(II) complexes were prepared and tested for inhibitory activity towards chlorophyll development of plants [18]. Finally, X-ray crystallographic studies of the complexes of a dinuclear nitrogen bridged tetronic acid with Cu(II) and Ni(II) [19] showed that Cu(II) coordinates by means of two nitrogen and two oxygen atoms of the ligand and one water molecule on the top of a tetragonal pyramid. In contrast, the Ni(II) complex, having an extra water molecule, forms a nearly regular octahedron structure.

In the course of our research program on the synthesis of five membered heterocyclic compounds, we have developed a new advantageous methodology for the synthetic approach of functionalized tetronic [20] and thiotetronic acids [21]. The common feature of these heterocycles is the *β*,*β*′-tricarbonyl system which provides them with sites available for metal complexation. Based upon the observation that tetramic acid analogues with metal ions show increased biological activities [22, 23], we have investigated the complexation of tetramic acids with several metal ions [24–29].

**Scheme 1:**
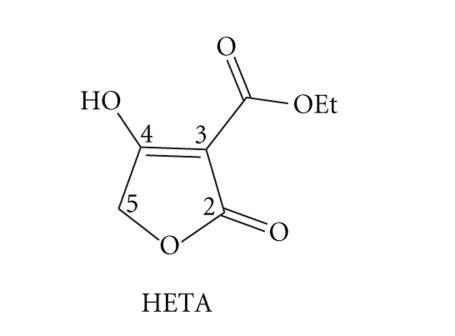
3-Ethoxycarbonyltetronic acid.

In this paper, we examined the complexation of 3-ethoxycarbonyltetronic acid (HETA) (Scheme 1) with Cu(II) and Co(II) ions. We report herein our results based on the data collected after EPR spectroscopy and magnetic susceptibility measurements, and using these data, we propose structures for these complexes.

## 2. EXPERIMENTAL

### 2.1. Materials and methods

Reagent grade chemicals and solvents (Fluka, Aldrich, Acros) were used without further purification unless otherwise noted. Infrared spectra were recorded in KBr in the range 4000–400 cm^−1^ on a Nicolet Magna 560R FT-IR spectrophotometer. C, H, and N analyses were performed in the Organic Chemistry Laboratory (NTUA) using a EuroVector EA 3000 elemental analyzer. ^1^H and ^13^C NMR spectra were recorded on a Varian Gemini-2000 300 MHz spectrometer. The magnetic susceptibility measurements were made using a Gouy balance at room temperature using mercury tetrathiocyanatocobaltate (II), Hg[Co(NCS)_4_] as calibrant. EPR measurements were obtained at 4.2 K with an upgraded Bruker ER-200D spectrometer interfaced to a personal computer and equipped with an Oxford ESR 900 cryostat, an Anritsu MF76A frequency counter, and a Bruker 035M NMR gaussmeter. The perpendicular 4102ST cavity was used, and the microwave frequency was 9.41 GHz. The samples used for the EPR measurements were powders.

#### 2.1.1. 3-Ethoxycarbonyl tetronic acid (HETA) [20]

 Powder (1.07 g, 62*%*), m.p. 112–114°C (*Anal*. Found: C, 48.82; H, 4.80. Calc. for C_7_H_8_O_5_: C, 48.63; H, 4.65*%*); *v*_max_/cm^−1^ (C=O) 1761, 1651, (C=C) 1605; *δ*_H_(DMSO‐*d*_6_) 1.20 (3H, t *J* = 7.5 Hz, COOCH_2_CH_3_), 4.14 (2H, q *J* = 7.5 Hz, COOCH_2_CH_3_), 4.68 (2H, s, CH_2_ ring); *δ*_C_(DMSO‐*d*_6_) 14.3 (COOCH_2_CH_3_), 59.2 (COOCH_2_CH_3_), 66.5 (C‐5), 91.9 (C‐3), 161.1 (C‐6), 169.5 (C‐2), 186.1 (C‐4).

#### 2.1.2. [Cu(ETA)(OAc)] (1) (Ac = acetyl)

 A methanolic solution (12 mL) of the ligand (2.5 mmol) was added to a refluxing methanol solution (30 mL) of Cu(OAc)_2_·H_2_O (2.5 mmol). The resulting solution was refluxed for 2 hours. The solution was evaporated to a small volume and the deposited precipitate was collected by filtration, washed with cold methanol, diethylether and dried in vacuo over P_2_O_5_. Powder (0.53 g, 72%), *μ*_eff_ 2.08 *μ*_B_; (*Anal*. Found: C, 36.82; H, 3.67. Calc. for C_9_H_10_O_7_Cu: C, 36.70; H, 3.41); *v*_max_/cm^−1^ (C=O and C=O) 1724s, 1634s, 1554s, 1497s, 1402m, (Cu−O) 525w, 477w.

#### 2.1.3. [Cu(ETA)(OAc)*·*H_*2*_O]_*2*_ (2) (Ac = acetyl)

 A methanolic solution (12 mL) of the ligand (1.9 mmol) was added to a refluxing methanol solution (15 mL) of Cu(OAc)_2_·H_2_O (0.95 mmol). The resulting solution was refluxed for 2 hours. The solution was evaporated up to a small volume of the solvent and the deposited precipitate was collected by filtration, washed with cold methanol, diethylether, and dried in vacuo over P_2_O_5_. Powder (0.25 g, 90%), *μ*_eff_ 1.63 *μ*_B_; (*Anal*. Found: C, 34.97; H, 3.67. Calc. for C_18_H_24_O_16_Cu_2_: C, 34.67; H, 3.85); *v*_max_/cm^−1^ (OH) 3542br, 3444br, (C=O and C=C) 1726s, 1635s, 1557s, 1499s, 1441m, 1403m, (Cu−O) 523w, 475w.

#### 2.1.4. [Cu(ETA)_*2*_ *·*(H_*2*_O)_*2*_] (3)

A methanolic solution (12 mL) of the ligand (2.0 mmol) was added to a refluxing methanol solution (10 mL) of CuCl_2_·2H_2_O (1.0 mmol). The resulting solution was refluxed for 2 hours. The solution was evaporated to a small volume and the deposited precipitate was collected by filtration, washed with cold methanol, diethylether, and dried in vacuo over P_2_O_5_. Light blue powder (0.27 g, 64%), *μ*_eff_ 2.17 *μ*_B_; (*Anal*. Found: C, 38.22; H, 3.78. Calc. for C_14_H_16_O_11_Cu: C, 38.04; H, 4.07); *v*_max_/cm^−1^ (OH) 3540br, 3436br, 3276br, (C=O and C=C) 1724s, 1635s, 1558s, 1498s, (Cu−O) 526w, 467w.

#### 2.1.5. [Co(ETA)(OAc)*·*CH_*3*_OH] (4) (Ac = acetyl)

 A methanolic solution (5 mL) of the ligand (1.3 mmol) was added to a refluxing methanol solution (10 mL) of Co(OAc)_2_·4H_2_O (1.3 mmol). The resulting solution was refluxed for 2 hours. The solution was evaporated to a minimal volume and the deposited precipitate was collected by filtration, washed with cold methanol, diethylether, and dried in vacuo over P_2_O_5_. Powder (0.26 g, 62%), *μ*_eff_ 4.96 *μ*_B_; (*Anal*. Found: C, 37.16; H, 4.04. Calc. for C_10_H_14_O_8_Co: C, 37.38; H, 4.36); *v*_max_/cm^−1^ (OH) 3466br, 3281br, (C=O and C=C) 1711s, 1651s, 1567s, 1492s, 1441m, 1400m, (Co‐O) 528w, 432w.

## 3. RESULTS AND DISCUSSION

**Scheme 2:**
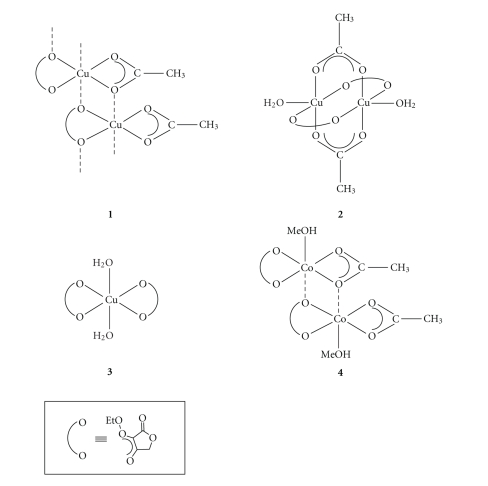


The complexes M_*u*_(OAc)_*x*_(ETA)_*y*_(H_2_O)_*z*_(MeOH)_*w*_ (where in **1**, M = Cu, *u* = 1, *x* = 1, *y* = 1, *z* = 0, *w* = 0;**2**, M = Cu, *u* = 2, *x* = 2, *y* = 2, *z* = 2, *w* = 0;**3**, M = Cu, *u* = 1, *x* = 0, *y* = 2, *z* = 2, *w* = 0;**4**, M = Co, *u* = 2, *x* = 2, *y* = 2, *z* = 0, *w* = 2 (Scheme 2) were prepared by reaction of the appropriate acetate salt M(OAc)_2_·*x*H_2_O (*x* = 1, and 4 for compounds **1**, **2**, **4**) or chloride salt MCl_2_·*x*H_2_O (for compound **3**, M = Cu, *x* = 2) and HETA in MeOH under reflux by simply changing the metal:ligand ratio.

The complexes were isolated as powders following evaporation of the mixture to a minimum volume. The products were stable in the normal laboratory atmosphere and soluble in warm MeOH. An interesting feature in the synthesis of complexes was the impossibility of isolating a complex of the “core formula” M(ETA)_2_ when we used M(OAc)_2_ *x*H_2_O:HETA in a 1:2 ratio; the only isolable compounds were M(OAc)(ETA) complexes.

The IR spectra of the metal complexes **1–** **4** show the *ν*(C=O) lactam and *ν*(C=O) diketone characteristic bands shifted to lower wave numbers with respect to those of the free ligands confirming that two oxygen atoms are involved in the coordination sphere of the metal [30]. New bands at higher frequencies (~3550, ~3450, ~3300 cm^−1^) appeared when the ligands were complexed to the metal ions (data not shown). These bands can be attributed to the stretching vibrations of the OH group from coordinated water or methanol molecules.

Since the IR spectra gave evidence that the metals were successfully complexed with HETA via oxygen atoms, we performed magnetic susceptibility studies to gain initial information about the spin states of the metal centers.The magnetic moments at room temperature of Cu(II) complexes **1**, **2** and **3** (2.08 BM, 1.63 BM, and 2.17 BM, resp.) indicated that no reduction to Cu(I) had occurred, whereas that of Co(II) complex **4** (4.96 BM) was characteristic of octahedral stereochemistry. The complexes **1** and **3** with d^9^ configuration of the central atom are magnetically diluted systems and they have the expected values [27]. However, for the complex **2**, with *μ*_eff_ = 1.63 BM, the observed value was somewhat less than the spin only value for an *S* = 1/2 system and noticeably less than that expected for a magnetically isolated Cu(II) system [31]. This behavior may be attributed to the presence of weak intermolecular interactivity (possibly involving a hydrogen bonding network), but variable temperature measurements will be required to quantify such effects [32]. The study of these effects is currently in progress in our laboratory.

**Figure 1:**
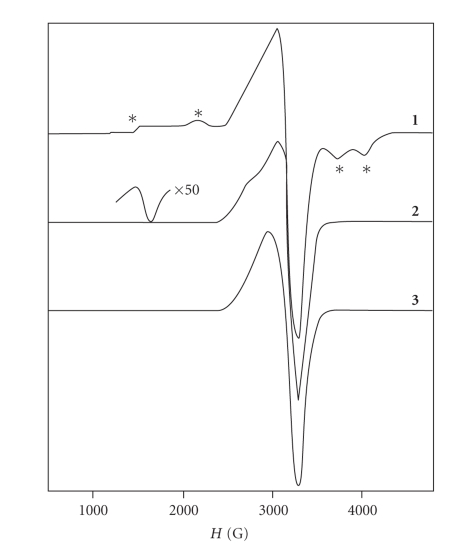
X-band spectrum of compounds **1**, **2**, and **3**. EPR conditions: microwave frequency 9.407 GHz, temperature 4.2 K, mod. ampl. 8 Gpp, microwave power 8.2 mW, sweep time 200 seconds, t.c.: 300 milliseconds. The asterisks (∗) indicate unique peaks characteristic for compound **1**.

The EPR spectra of compounds **1**, **2** and **3** were recorded at 4.2 K (Figure 1). For compound **1**, the spectrum exhibited two sets of signals. The first set consisted of four peaks at 1636G, 2170G, 3715G, and 4010G (indicated by asterisks, Figure 1), while the second signal defined an asymmetric feature at the region 2507–3528G.

The presence of the first set of signals indicates the population of an *S* = 1 triplet state which is characteristic of dinuclear Cu(II) systems [33]. The spin Hamiltonian for a triplet state is given by the following equation [34]: H^=g⋅μB⋅H⋅S^+D⋅[Sz2−(1/3)⋅s⋅(s+1)]+E⋅(Sx2−Sy2).

Here, *D* and *E* are the zero field splitting parameters, *μ*_*B*_ is the Bohr magneton, and *x*, *y*, *z* are the principal axes. According to Wasson et al. [35] for the case of a rhombic symmetry (*E* ≠ 0), two transitions are allowed by the transition rule (Δ*m*_*s*_ = ±1) along each principal direction, and therefore six resonance fields can be determined. In axial symmetry (*E* = 0), four Δ*m*_*s*_ = ±1 transitions are allowed. These four resonance fields are given by the following equations:

(1) $$\begin{array}{ll} H_{z^{1}} & =\left(\frac{g_{e}}{g_{z}}\right)\cdot (H_{0}-D^{\prime }),\\ H_{xy^{1}}^{2} & ={\left(\frac{g_{e}}{g_{xy}}\right)}^{2}\cdot H_{0}\cdot \left(H_{0}-D^{\prime }\right),\\ H_{xy^{2}}^{2} & ={\left(\frac{g_{e}}{g_{xy}}\right)}^{2}\cdot H_{0}\cdot (H_{0}+D^{\prime }),\\ H_{z^{2}} & =\left(\frac{g_{e}}{g_{z}}\right)\cdot \left(H_{0}+D^{\prime }\right), \end{array}$$

where *H*_0_ = *h*·*ν*/*g*·*μ*_*B*_ and *D*′ = *D*/*g*·*μ*_*B*_.

The signals centered at 1636G, 2170G, 3715G, and 4014G, assigned to the resonance fields *H*_*z*^1^_ *H*_*xy*^1^_ *H*_*xy*^2^_ *H*_*z*^2^_, respectively, are indicative of copper species in a tetragonally distorted octahedral environment. From the above equations of four Δ*m*_*s*_ = ±1 transitions, the calculated parameters are the following: *D* = 0.13 cm^−1^, *g*_*z*_ = 2.35, *g*_*xy*_ = 2.2. At higher temperatures, the four resonance fields disappeared (data not shown). These effects, together with the small magnitude of the *D* value, indicated weak interactions (mainly dipolar) between Cu⋯Cu ions. However, the features at the region 2507–3528G consisted of a derivative at *g* ~2.13 and a shoulder at *g* ~2.45. This set of signals was characteristic of a spin doublet S = 1/2 with an axial symmetry. We assigned these signals to mononuclear Cu(II) species in a tetragonally distorted octahedral environment with *g*_//_ = 2.45 and *g*_⊥_ = 2.13. The fact that *g*_//_ > *g*_⊥_ was consistent with a *d*_*x*^2^-*y*^2^_ orbital ground state of the copper ion.

The spectrum of compound **2** consists mainly of a derivative line at *g* ~2.1, and an absorption peak at *g* ~2.5. We assigned these features to an axial *S* = 1/2 species with *g*_//_ = 2.5 and *g*_⊥_ = 2.1, attributed to a monomer copper center. The fact that *g*_//_ > *g*_⊥_ suggests that the unpaired electron is localized in the *d*_*x*^2^-*y*^2^_ orbital. Moreover, the presence of a weak signal at ~1600G, often encountered in several copper complexes [36], is attributed to Δ*m*_*s*_ = ±2 transition, owing to *S* = 1 triplet state population. This signal indicated the existence of the dinuclear species of this copper compound.

The EPR spectrum from compound **3** exhibited an asymmetric feature at the region 2330–3650G, which was assigned to a mononuclear copper complex. The asymmetric form of this signal indicated g anisotropy, with *g*_⊥_ = 2.1. The *g*_//_ component is not well resolved. The absence of signals characteristic for the S = 1 population indicated that dinuclear species of this compound does not exist.

**Figure 2:**
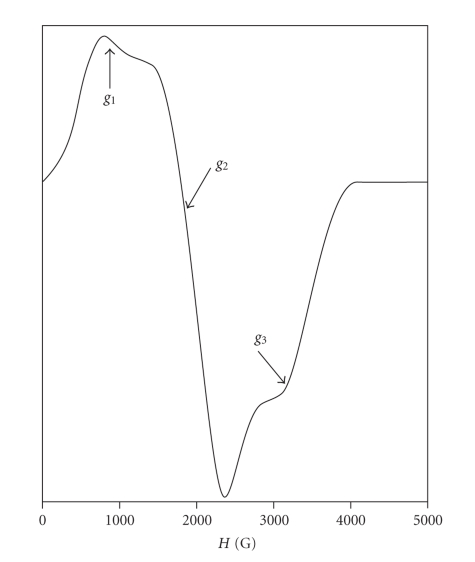
X-band spectrum of compound **4**. EPR conditions: microwave frequency 9.407 GHz, temperature 4.2 K, mod. ampl. 8 Gpp, microwave power 0.6 mW, sweep time 200 seconds, t.c.: 300 milliseconds. The arrows indicate regions of *g* anisotropy.

The EPR spectrum of compound **4**, recorded at 4.2 K, is dominated by one resonance derivative line at *g*_2_ = 3.5, as well as signals at *g*_1_ = 8.3 and *g*_3_ = 2.17 (Figure 2). These signals are characteristics of high-spin (*S* = 3/2) cobalt ion, whose spectroscopic states are separated. The splitting of spectroscopic states of a d^7^ configuration in coordination complexes results in two general patterns, either in an orbitally nondegenerate ground state (^4^A_2_), as may be found in tetra- and penta-coordinate sites, or in an orbitally degenerated ground state (^4^T_1_) in which the orbital levels are separated by spin-orbit coupling, as in cases of high symmetry crystal field [37]. The magnetic moment of Co(II) for compound **4** (4.96 *μ*_B_) is characteristic of octahedral stereochemistry, suggesting that its ground state is the orbitally degenerate ^4^T_1_. The combined effects of spin-orbit coupling and distortion of the crystal field from high symmetry lead to a series of Kramer doublets *m*_*s*_ = ±1/2, *m*_*s*_ = ±3/2. The ^4^T_1_ ground state is split into a series of levels approximately described by fictitious orbital angular momentum *L* = 1 and the corresponding *J* values of 1/2(*m*_*j*_ = ±1/2), 3/2 (*m*_*j*_ = ±1/2, ±3/2), and 5/2(*m*_*j*_ = ±1/2, ±3/2, ±5/2). The presence of the three *g* values of the EPR spectrum indicated *g* anisotropy, which may be assigned to the above combined effects.

## 4. CONCLUSIONS

The isolated complexes of Cu(II) and Co(II) acetates with HETA in 1:1 ratio have octahedral stereochemistry with bidentate co-ordination through O(4) and O(6) of the tetronate ring and structures of the general formula M(OAc)_*x*_(ETA)_*y*_(H_2_O)_*z*_(MeOH)_*w*_. The chloride Cu(II) complex **3** with HETA in 1:2 ratio has octahedral stereochemistry and a structure of the formula M(ETA)_2_·2H_2_O. Both Cu(II) complexes **1** and **2** showed the presence of two sets of EPR signals indicating an inhomogenity of centers; some of them point to a mononuclear structure, while the others adopt a dinuclear structure [38]. Moreover, EPR studies for compounds **3** and **4** showed mononuclear and dinuclear structures, respectively. In summary, we have prepared a plausible model for the copper-cobalt *β*,*β*′-tricarbonyl coordination compounds. Our proposed model may help define some of the unusual features associated with copper and cobalt metallobiochemistry.
